# Autophagic Turnover of Chloroplasts: Its Roles and Regulatory Mechanisms in Response to Sugar Starvation

**DOI:** 10.3389/fpls.2019.00280

**Published:** 2019-03-22

**Authors:** Masanori Izumi, Sakuya Nakamura, Nan Li

**Affiliations:** ^1^ Frontier Research Institute for Interdisciplinary Sciences, Tohoku University, Sendai, Japan; ^2^ Department of Molecular and Chemical Life Sciences, Graduate School of Life Sciences, Tohoku University, Sendai, Japan; ^3^ PRESTO, Japan Science and Technology Agency, Kawaguchi, Japan; ^4^ College of Life Sciences, Liaocheng University, Liaocheng, China

**Keywords:** amino acid recycle, autophagy, catabolism, chloroplast, Rubisco-containing body, sugar starvation

## Abstract

Photosynthetic reactions in chloroplasts convert atmospheric carbon dioxide into starch and soluble sugars during the day. Starch, a transient storage form of sugar, is broken down into sugars as a source for respiratory energy production at night. Chloroplasts thus serve as the main sites of sugar production for photoautotrophic plant growth. Autophagy is an evolutionarily conserved intracellular process in eukaryotes that degrades organelles and proteins. Numerous studies have shown that autophagy is actively induced in sugar-starved plants. When photosynthetic sugar production is inhibited by environmental cues, chloroplasts themselves may become an attractive alternative energy source to sugars *via* their degradation. Here, we summarize the process of autophagic turnover of chloroplasts and its roles in plants in response to sugar starvation. We hypothesize that piecemeal-type chloroplast autophagy is specifically activated in plants in response to sugar starvation.

## Introduction

Living organisms require energy for their growth and survival. In plants and algae, the chloroplast serves as the primary site for energy production *via* photosynthesis. This process involves the photosynthetic conversion of sunlight energy into forms of chemical energy such as ATP and NADPH, which in turn drive the assimilation of atmospheric CO_2_ for sugar production. Therefore, when photosynthetic energy production within chloroplasts is impaired, plants experience sugar starvation (Smith and Stitt, 2007). In addition to insufficient light due to suboptimal climate conditions or shading by neighboring plants, some abiotic stresses such as extreme temperature, drought, and submergence also impair photosynthetic sugar production, thereby inducing sugar starvation (Baena-González and Sheen, 2008; McDowell et al., 2008). Therefore, understanding how plants adapt to sugar starvation is important for developing new avenues for improving abiotic stress tolerance in crops.

A simple, widely used experimental system for inducing sugar starvation involves exposing whole plants to complete darkness for several days (Weaver and Amasino, 2001; Keech et al., 2007). Large-scale transcriptome and metabolome analyses of dark-treated *Arabidopsis* (*Arabidopsis thaliana*) plants have been performed to evaluate the various responses to sugar starvation (Buchanan-Wollaston et al., 2005; Usadel et al., 2008; Law et al., 2018). These studies have revealed that catabolic processes that degrade previously synthesized cellular components are important for adaptation to sugar starvation, since catabolism can produce alternative energy sources to photosynthesis-derived sugars. Autophagy is an evolutionarily conserved degradation process for intracellular components that facilitates catabolic reactions in eukaryotic cells. Recent studies have uncovered several autophagic pathways that degrade chloroplasts in plant cells. In this mini-review, we focus on the autophagic pathways that degrade chloroplasts, their roles, and their possible regulatory mechanisms during sugar starvation.

## Brief Overview of the Autophagy Machinery in Yeast

Molecular genetic studies in the budding yeast *Saccharomyces cerevisiae* have uncovered the basic machinery used for autophagic turnover of intracellular components (Nakatogawa et al., 2009). During the most well-characterized autophagic process, macroautophagy (Feng et al., 2014), newly synthesized, double-membrane-bound vesicles termed autophagosomes engulf a portion of the cytoplasm and deliver their cargo to the vacuole. The inner-membrane-bound structures of autophagosomes, termed autophagic bodies, are released into the vacuolar lumen for degradation. To date, over 40 genes related to autophagy (*ATGs*) have been identified in *S. cerevisiae*. Of these, 15 “core” ATGs (ATG1–10, ATG12–14, ATG16, and ATG18) are considered the fundamental players for the biogenesis of autophagosomal membranes (Nakatogawa et al., 2009). ATG3–5, ATG7, ATG10, ATG12, and ATG16 participate in producing the conjugated form of the ubiquitin-like ATG8 protein with the lipid, phosphatidylethanolamine (PE), which helps build the nascent autophagosomal membrane (Mizushima et al., 1998; Mizushima et al., 1999; Ichimura et al., 2000; Suzuki et al., 2001). Core *ATG* orthologs are largely conserved in plants; mutant analysis of core *ATGs* in *Arabidopsis* demonstrated that the machinery required for autophagosome formation is similar to that of *S. cerevisiae*, as described in recent reviews (Soto-Burgos et al., 2018; Yoshimoto and Ohsumi, 2018; Marshall and Vierstra, 2018a).

In yeast, ATG11 is not classified as a core ATG because of its dispensability for starvation-induced production of autophagosomes that facilitate bulk digestion of cytoplasm (Nakatogawa et al., 2009). However, nitrogen starvation-induced macroautophagy is impaired in *Arabidopsis atg11* mutant plants (Li et al., 2014). Furthermore, *Arabidopsis* ATG11 is a component of the ATG1–ATG13 kinase complex (Li et al., 2014), which is an essential, upstream regulator of macroautophagy. Therefore, plant ATG11 may act as a core ATG protein similar to mammalian FIP200. In mammals, the counterpart of ATG1 kinase complex comprises ULK1/2, ATG13, FIP200, and ATG101 (Hara et al., 2008; Hosokawa et al., 2009).

Microautophagy involves the vacuolar transport of cytoplasmic components through direct sequestration by the vacuolar membrane (Oku and Sakai, 2018). In the methylotrophic yeast *Komagataella phaffii* (formerly known as *Pichia pastoris*), multiple peroxisomes are incorporated into the vacuole *via* a microautophagic process when the energy source for growth switches from methanol to glucose (Mukaiyama et al., 2004). This micropexophagy process in *K. phaffii* requires the activities of core ATG proteins (Oku and Sakai, 2018). A microautophagic process that degrades lipid droplets termed microlipophagy has been described in *S. cerevisiae* (Vevea et al., 2015). This pathway occurs independently of core ATG proteins but requires the Endosomal Sorting Complex Required for Transport (ESCRT) machinery for endosomal membrane remodeling (Vevea et al., 2015; Oku et al., 2017). Therefore, in addition to core ATG-dependent microautophagy, ESCRT-dependent microautophagy also functions in yeast.

## Sugar Starvation Induces Piecemeal Degradation of Chloroplasts by Autophagy

Free amino acids (AAs) derived from protein degradation represent an important alternative energy source during sugar starvation (Araújo et al., 2011). Chloroplasts in leaf mesophyll cells contain most of the proteins needed to construct the photosynthetic apparatus; the CO_2_-fixing enzyme Rubisco accounts for approximately 50% of total soluble proteins in the leaves of C3 plants (Makino and Osmond, 1991). The high amount of protein in chloroplasts makes them an attractive target for degradation to provide free AAs as an alternative to sugars.

**Figure 1:**
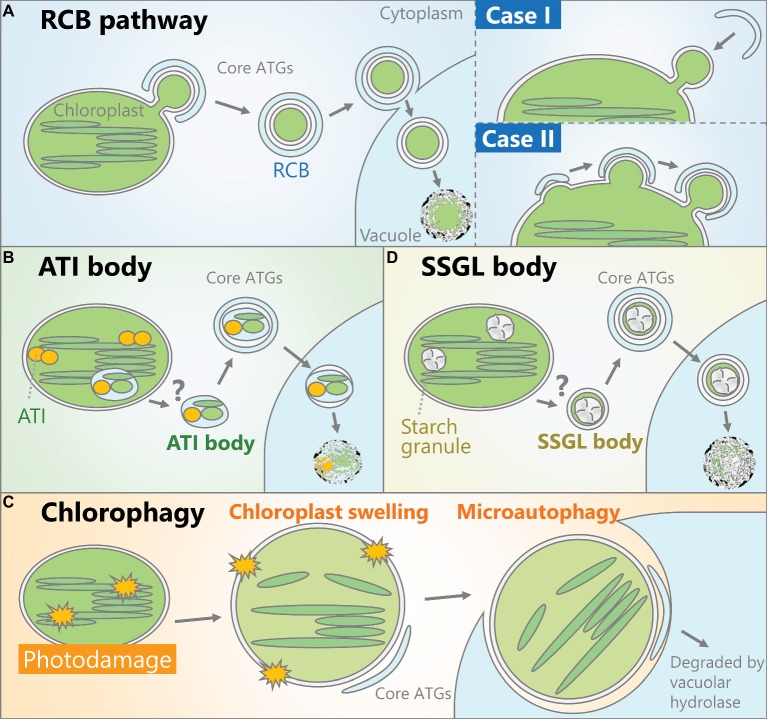
Schematic models of the autophagic pathways that degrade chloroplasts. **(A)** A portion of the chloroplast is delivered into the vacuolar lumen as an autophagosome vesicle containing stromal components including Rubisco, termed a Rubisco-containing body (RCB). How such autophagosomes are produced remains unclear. One possibility (Case I) is that the protruded parts of chloroplasts such as stromules are recognized by the autophagic membrane and engulfed as autophagosomes. Alternatively (Case II), the autophagic membrane might interact with part of the chloroplast, followed by the concurrent release of RCBs and the maturation of autophagosomes. **(B)** Chloroplast-associated bodies containing ATG8-interacting protein 1 (ATI1) and ATI2 are generated inside the chloroplast and transport proteins in the stroma, thylakoid, and chloroplast envelope into the vacuolar lumen for degradation by autophagy. **(C)** When chloroplasts are exposed to severe damage, such as irradiation by strong visible light, some chloroplasts exhibit swelling due to envelope damage and the subsequent osmotic imbalance between the stroma and cytoplasm. These damaged chloroplasts are entirely engulfed by the vacuolar membrane to be selectively incorporated into the vacuolar lumen. **(D)** Starch granules, the transient storage form of photoassimilate, can be transported as small starch granule-like structures (SSGLs) engulfed by autophagosomal membrane.

A route for the degradation of stromal proteins including Rubisco has been proposed that does not involve the complete digestion of chloroplasts (Mae et al., 1983; Ono et al., 1995). Immunoelectron microscopy analysis using an anti-Rubisco antibody revealed small vesicles containing Rubisco in wheat (*Triticum aestivum*) leaves (Chiba et al., 2003). These ~1-μm-diameter cytoplasmic vesicles are referred to as Rubisco-containing bodies (RCBs). More recent studies visualized these vesicles *in vivo* in *Arabidopsis* leaves using stroma-targeted fluorescent proteins and fluorescent protein-labeled Rubisco (Ishida et al., 2008) and confirmed that RCBs are transported into the vacuole. RCBs are not produced in mutants of core *ATGs* such as *atg4* and *atg5* and can be decorated by the autophagosomal membrane marker green fluorescent protein (GFP)-ATG8 in the vacuole (Ishida et al., 2008; Wada et al., 2009; Ono et al., 2013). These studies uncovered an autophagic process that degrades chloroplast stroma, termed the RCB pathway (Figure 1A). This pathway also has been described in rice (*Oryza sativa*) leaves (Izumi et al., 2015).

In *S. cerevisiae*, ATG8 binds to specific proteins and delivers them as cargoes of autophagosomes (Noda et al., 2010). Organelles including mitochondria, the endoplasmic reticulum (ER), and nuclei can become targets of autophagy through interactions between ATG8 and each organelle-localized ATG8-interacting protein (Kanki et al., 2009; Okamoto et al., 2009; Mochida et al., 2015). ATG8-interacting protein 1 (ATI1) and ATI2 were identified in *Arabidopsis via* yeast two-hybrid screening of ATG8-interacting partners (Honig et al., 2012). Interactions between those ATIs and the ATG8 family member ATG8f were observed *in vivo* in a bimolecular fluorescence complementation assay. In ATI1-GFP-expressing plants, small vesicles (~1 μm in diameter) exhibiting ATI1-GFP signals were observed, which interacted with the ER or chloroplasts (Michaeli et al., 2014). ATI1 can bind to some proteins in the chloroplast stroma, thylakoid, and envelope, and the emergence of ATI1-GFP-labeled bodies from chloroplasts has been observed. Such bodies are transported into the vacuolar lumen in wild-type plants, but not in the *atg5* mutant. Therefore, the vacuolar transport of chloroplast segments with ATI bodies is an *ATG*-dependent autophagic process that degrades proteins in the chloroplast stroma, thylakoid, and envelope (Figure 1B; Michaeli et al., 2016), although how such bodies sequester a portion of the thylakoid and emerge from chloroplasts remains uncertain (Figure 1B). The authors who detected chloroplast-associated ATI bodies suggested that these bodies do not contain Rubisco (Michaeli et al., 2014). Since the cargoes of RCBs and ATI bodies are not the same, these autophagic vesicles are considered to function in distinct pathways (Michaeli et al., 2014; Izumi and Nakamura, 2018).

Chloroplasts accumulate a portion of their photoassimilate as insoluble starch granules during the day, and degrade them to generate soluble sugars for respiratory energy production during the night (Smith and Stitt, 2007). In *Arabidopsis* mesophyll cells, starch breakdown occurs in chloroplasts through several enzymatic reactions and deficiencies in starch-degrading enzyme cause hyperaccumulation of starch (Zeeman et al., 2010). Notably, a study mainly using *Nicotiana benthamiana* leaves showed the participation of autophagy in this starch granule degradation (Wang et al., 2013). Electron microscopy observation and the behavior of fluorescent protein-tagged GRANULE-BOUND STARCH SYNTHASE I, a fluorescent marker of starch granules, indicated the vacuolar transport of starch granules by a type of autophagosome during the night. In addition, virus-induced gene silencing of some *ATG* genes led to an excess accumulation of starch (Wang et al., 2013). Thus, autophagy contributes to the degradation of starch granule-containing vesicles termed small starch granule-like structures (SSGLs; Figure 1C).

In excised *Arabidopsis* rosette leaves, RCB production is activated in the dark, but not in the light (Izumi et al., 2010). Even in darkness, the addition of exogenous sucrose suppresses RCB accumulation. A similar phenomenon was detected in rice leaves (Izumi et al., 2015). In mutants of enzymes that synthesize starch from sugars, more severe sugar starvation occurs in darkness due to the absence of the storage form of sugars (Smith and Stitt, 2007). More RCBs are produced in starchless mutants than in wild-type plants (Izumi et al., 2010; Izumi et al., 2013). These findings indicate that the RCB pathway is actively induced during sugar starvation. The appearance of plastid-associated ATI bodies is also induced in the cotyledons of plants maintained in darkness for 1–3 days (Michaeli et al., 2014). Overall, the piecemeal degradation of chloroplast proteins by autophagy is likely induced in response to sugar starvation.

## Chlorophagy Degrades Whole Chloroplasts

Whole chloroplasts are also subjected to autophagy, as recent studies have indicated that damaged whole chloroplasts are removed by an autophagic process termed chlorophagy (Nakamura and Izumi, 2018). In plants subjected to photodamage *via* exposure to strong visible light, ultraviolet-B, or natural sunlight, whole chloroplasts, including stroma and thylakoids, are transported into the vacuolar lumen (Izumi et al., 2017). This phenomenon does not occur in mutants of *ATG5* or *ATG7*; therefore, chlorophagy is dependent on the core *ATG* genes.

After exposure to strong visible light, a subset of chloroplasts in a single mesophyll cell exhibits swelling, which is likely caused by envelope damage and the resulting imbalance of osmotic pressure across the envelope (Nakamura et al., 2018). In wild-type plants, swollen chloroplasts are partially labeled by GFP-ATG8 and entirely engulfed by the vacuolar membrane for transfer into the vacuolar lumen. Thus, chlorophagy is a selective autophagic pathway that removes chloroplasts with damaged envelopes *via* a microautophagic process (Figure 1D).

## Increased Free AA Supply *via* Autophagy Helps Plants Overcome Sugar Starvation

Numerous studies have shown that *Arabidopsis* mutants of the core *ATG* genes exhibit accelerated cell death and reduced survival during dark treatment compared to wild-type plants (Doelling et al., 2002; Hanaoka et al., 2002; Thompson et al., 2005; Xiong et al., 2005; Phillips et al., 2008; Chung et al., 2010; Suttangkakul et al., 2011), indicating that autophagy plays an essential role in plant adaptation to sugar starvation. The elongation of etiolated seedlings that have been germinated and grown in darkness is retarded in *atg* plants; thus, autophagy is required for the heterotrophic growth of germinated seedlings until they acquire light energy and begin photoautotrophic growth (Avin-Wittenberg et al., 2015). Free AA levels are lower in etiolated seedlings of *atg* versus wild-type plants, indicating that autophagic protein turnover releases free AAs to support the growth of etiolated seedlings.

Recent studies have measured the changes in free AA levels in mature *Arabidopsis* rosette leaves during dark treatment to assess the importance of autophagy in the responses to sugar starvation (Barros et al., 2017; Hirota et al., 2018). Although the overall levels of free AAs were higher in the leaves of dark-treated plants, the increase in basic AA (Lys, Arg, and His), aromatic AA (Phe, Tyr, and Trp), and branched-chain amino acid (BCAA; Val, Leu, and Ile) levels was compromised in *atg* plants relative to the wild type. Thus, autophagy plays a vital role in the production of free AAs when photosynthesis is impaired in mature plants.

The levels of free basic AAs, BCAAs, and aromatic AAs are extremely low in plants grown under a light/dark cycle without stress treatment (Hildebrandt et al., 2015). Numerous metabolomic analyses have uncovered a drastic increase in free basic AA, BCAA, and aromatic AA levels in plants subjected to sugar starvation due to dark treatment (Ishizaki et al., 2005; Ishizaki et al., 2006; Araújo et al., 2010; Peng et al., 2015; Barros et al., 2017; Hirota et al., 2018; Law et al., 2018). Under such conditions, free AAs are further catabolized through enzymatic cascades into simple carbon skeletons that can be integrated into the mitochondrial TCA cycle for respiratory energy production (Hildebrandt et al., 2015). Mutants of enzymes that catabolize BCAAs in mitochondria show hyperaccumulation of BCAA and a reduced survival rate during dark treatment (Ishizaki et al., 2005; Ishizaki et al., 2006; Araújo et al., 2010; Peng et al., 2015), indicating that BCAA catabolism plays an essential role in energy production during sugar starvation. As mentioned above, *atg* plants are also highly susceptible to dark treatment (Doelling et al., 2002; Phillips et al., 2008). The similar phenotypes among mutants of autophagy and BCAA catabolic enzymes during dark treatment point to a route for alternative energy production in which autophagic protein degradation supplies free BCAAs to the catabolic cascade within mitochondria. In fact, the enhanced accumulation of free BCAAs observed in single mutants of BCAA catabolism-related genes *ELECTRON TRANSFER FLAVOPROTEIN-UBIQUINONE OXIDOREDUCTASE* (*ETFQO*) and *ISOVALERYL-CoA DEHYDROGENASE* (*IVDH*) was diminished by the addition of the *atg5* mutation in double mutants (Hirota et al., 2018).

In mature *Arabidopsis* leaves, RCB production is activated during the early stage of dark treatment when plants are maintained in darkness for 2 days (Hirota et al., 2018). Consistent with this observation, under this treatment, wild-type plants showed a decrease in Rubisco and soluble protein levels and an increase in free AAs, whereas in *atg2* and *atg5*, Rubisco levels remained unchanged and the increase in free AAs was suppressed (Hirota et al., 2018). Chlorophagy did not occur in plants that were maintained in darkness for only 2 days, suggesting that piecemeal degradation of the chloroplast stroma *via* RCBs is a major route for free AA production during the initial stage of adaptation to sugar starvation. However, free BCAA accumulation is not completely suppressed in dark-treated *atg* plants (Barros et al., 2017; Hirota et al., 2018), suggesting that autophagy-independent proteolytic systems contribute to the free AA supply. The cytoplasmic 26S proteasome system degrades ubiquitinated proteins, functioning as a ubiquitous protein turnover system in eukaryotes (Vierstra, 2009). Although the 26S proteasome complex becomes a target of autophagic turnover during either nitrogen starvation or proteasome inhibition (Marshall et al., 2015), this pathway is inactive in root cells during sugar starvation (Marshall and Vierstra, 2018b). Therefore, the ubiquitin-proteasome system may represent another route for free AA production in response to sugar starvation.

During later stages of dark treatment (such as 2–9 days of complete darkness), autophagy-deficient mutants exhibit more free AA accumulation, enhanced rates of dark respiration, and drastic decreases in proteins and chlorophyll levels compared to wild-type plants (Barros et al., 2017; Hirota et al., 2018), suggesting that uncontrolled catabolic responses to sugar starvation are induced in these plants. In fact, *CHLOROPLAST VESICULATION* (*CV*), which induces autophagy-independent chloroplast degradation mediated by CV-containing vesicles (Wang and Blumwald, 2014), is strongly upregulated in dark-treated *atg* plants (Barros et al., 2017). Such unexpected responses due to a deficiency in autophagy likely leads to the accelerated loss of chlorophyll and proteins as well as cell death in these plants, as overexpression of *CV* induces cell death in *Arabidopsis* leaves (Wang and Blumwald, 2014).

**Figure 2:**
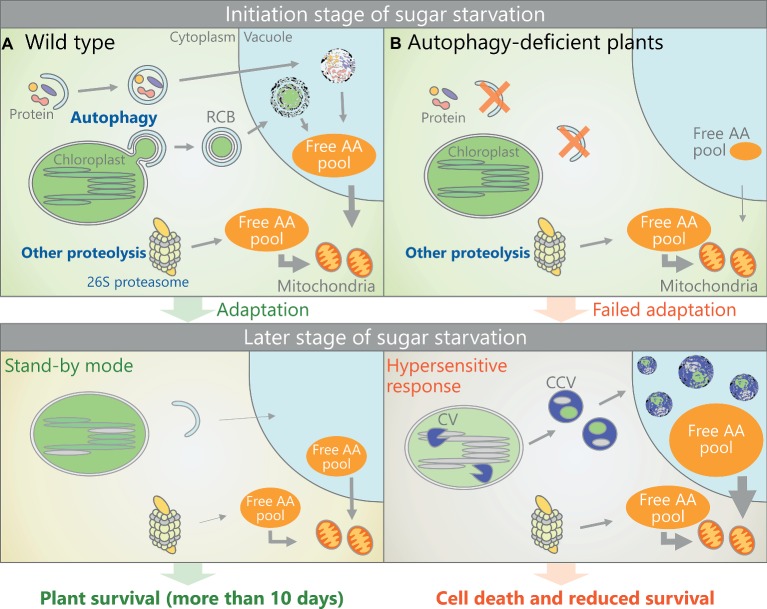
Schematic representation of a possible response to sugar starvation in *Arabidopsis* mesophyll cells. **(A)** In sugar-starved wild-type plants, autophagic degradation of the chloroplast stroma or other cytoplasmic components is activated, thereby increasing the free AA pool to help the plant adapt to sugar-starved conditions. This adaptive response allows plants to use a “stand-by mode” that prevents catabolic reactions from going into overdrive and to wait more than 10 days for environmental conditions to improve. **(B)** In sugar-starved *atg* plants, autophagy-mediated AA production (as an early response to sugar starvation) is impaired, thereby inducing a subsequent hypersensitive response to sugar starvation, including the strong activation of CV-containing vesicle (CCV)-mediated chloroplast degradation. Therefore, the *atg* plants cannot survive under long-term sugar starvation.

Figure 2 shows a schematic model of the possible responses of wild-type and *atg* plants to dark treatment. In wild-type plants, autophagic degradation of the chloroplast stroma and other components is activated as an early response to sugar starvation, leading to an increase in the free AA pool for respiratory energy production within the mitochondria and helping plants adapt to the sugar shortage (Figure 2A). Accordingly, wild-type plants can survive and “wait” more than 10 days for an improvement in the growth environment in “stand-by mode.” This hypothesis is consistent with the finding that protein and chloroplast degradation is suppressed during a later stage of dark treatment (Weaver and Amasino, 2001; Hirota et al., 2018; Law et al., 2018). The importance of ATI bodies in mature plants under sugar starvation remains unclear, as ATI body production in rosette leaves of dark-treated plants has not been assessed simultaneously with changes in protein or free AA levels. *Arabidopsis* leaves consume almost all of their starch by dawn during light/dark cycles (Smith and Stitt, 2007), indicating that starch breakdown is not sufficient to serve as the main response to sugar starvation induced by extended dark treatment (Izumi et al., 2013; Hirota et al., 2018). Therefore, autophagic degradation of SSGLs likely does not contribute to alleviate sugar starvation. By contrast, *atg* plants fail to produce a sufficient free AA pool during the initial stage of sugar starvation, leading to the hypersensitive response of autophagy-independent proteolysis during the later period, thereby resulting in accelerated cell death and a reduced survival rate (Figure 2B).

## Transcriptional Regulation of Core *ATGs* in Response to Sugar Starvation

SnRK1 (sucrose-non-fermentation1-related protein kinase1) acts as a central energy sensor that regulates global changes in gene expression in response to energy shortage in eukaryotes (Wurzinger et al., 2018). The transient overexpression of an *Arabidopsis* SnRK1 gene, *KIN10*, leads to increased transcription of various *ATG* and BCAA catabolism genes (Baena-González et al., 2007). KIN10 controls autophagosome production *in vivo*, as recently revealed using root cells of *kin10* and *KIN10*-overexpressing plants (Soto-Burgos and Bassham, 2017). ARABIDOPSIS TRANSCRIPTION ACTIVATION FACTOR1 (ATAF1) controls the transcription of both *ATG*s and BCAA catabolism genes during dark treatment (Garapati et al., 2015). Therefore, in addition to BCAA catabolism, the induction of autophagy is upregulated at transcriptional levels in response to sugar starvation.

Previous studies assessed how ATAF1 and SnRK1 coordinate during sugar starvation. ATAF1 and KIN10 interact directly (Kleinow et al., 2009), suggesting that there could be direct control of ATAF1 activity by the KIN10 kinase. By contrast, the results of several studies indicate that SnRK1 activity might be indirectly regulated by ATAF1. ATAF1 appears to influence the levels of trehalose-6-phosphate (Tre6P), as the overexpression of *ATAF1* reduces Tre6P contents (Garapati et al., 2015). Tre6P is a signaling molecule reflecting sugar status in plants (Figueroa and Lunn, 2016), and it is an inhibitor of *Arabidopsis* SnRK1 activity (Zhang et al., 2009). Notably, the production of autophagic vesicles during nitrogen starvation or abiotic stresses is suppressed by the addition of exogenous Tre6P in *Arabidopsis* seedlings (Soto-Burgos and Bassham, 2017). Thus, the changes of Tre6P status mediated by the function of ATAF1 may regulate KIN10 (a SnRK1) activity and the subsequent upregulation of ATG transcripts.

Target of rapamycin complex 1 (TORC1), an evolutionarily conserved kinase complex in eukaryotes, is a major upstream suppressor of autophagy that functions by phosphorylating the ATG1 complex; the TOR inhibitor rapamycin is widely used to mimic starvation and induce autophagy in *S. cerevisiae* (Noda, 2017). *Arabidopsis* TOR kinase also acts as a negative regulator of autophagy, as evidenced by an enhanced production of autophagic vesicles under non-stressed conditions in TOR-RNAi plants (Liu and Bassham, 2010). Whereas KIN10 overexpression activates autophagosome production, this effect is countered by additional TOR overexpression (Soto-Burgos and Bassham, 2017), indicating that KIN10 suppresses TOR activity to activate autophagy. As in yeast and mammals, the SnRK1-mediated suppression of TORC1 activity is crucial for activating the core ATG machinery in plants (Schepetilnikov and Ryabova, 2018; Soto-Burgos et al., 2018). Taken together, we hypothesize a possible regulatory route of ATG machinery during sugar starvation, in which the decrease in Tre6P level due to sugar starvation activates SnRK1, thereby leading to upregulation of ATG transcripts and inhibition of TORC1 to stimulate autophagosome production.

## Possible Regulatory Mechanism for Piecemeal Autophagy of the Chloroplast Stroma

In *S. cerevisiae*, the autophagic turnover of the perinuclear and cortical ER is specifically controlled by ATG39 and 40, respectively; these autophagic receptors bind to ATG8, even during starvation-induced autophagy (Mochida et al., 2015). An important issue in understanding the regulatory mechanism of chloroplast-targeting autophagy is whether piecemeal autophagy of the chloroplast stroma *via* RCBs is specifically regulated by a mechanism that does not involve the core autophagy machinery during sugar starvation. In detached *Arabidopsis* leaves, light irradiation specifically suppresses RCB production, whereas exogenous sugars suppress both autophagosome formation and RCB production (Izumi et al., 2010), suggesting that sugars endogenously produced *via* photosynthesis specifically attenuate RCB production without inhibiting the core autophagy machinery. Therefore, we propose that RCB production is specifically controlled in response to sugar starvation.

We predict two possible regulatory mechanisms that specifically control RCB production. First, morphological changes in chloroplasts might control their own autophagic degradation, since a portion of the stroma must protrude outward into the cytoplasm in order to be transported as a vesicle (Case I in Figure 1A). In *atg* plants, tubular extensions of plastids termed stromules are actively produced (Ishida et al., 2008). In *Arabidopsis* root cells, in which nonphotosynthetic plastids form numerous stromules (Hanson and Sattarzadeh, 2008), autophagic membranes interact with the edges of stromules (Spitzer et al., 2015). Therefore, a protrusion of tube-like structures may be required for chloroplasts to be separated as RCBs; the suppression of such morphological changes could inhibit RCB production, even when autophagosome production is activated. During autophagic degradation of mitochondria in mammalian cells, the small, circular mitochondria that are released from the division and fission cycles of mitochondria can become enclosed by autophagosomes (Rambold et al., 2011). Thus, morphological changes in organelles might be an important step that controls the occurrence of organelle-targeting autophagy.

However, unlike mitochondria (Arimura, 2018), mature leaf chloroplasts do not divide or undergo repeated fission, and they rarely form stromules (Hanson and Sattarzadeh, 2008). In yeast and mammalian cells undergoing mitophagy, the division of mitochondria destined for transport by autophagosomes occurs simultaneously with mitochondria-associated autophagosome formation (Yamashita et al., 2016). Therefore, it is conceivable that the association of the autophagic membrane on the chloroplast surface is the initial event that enables the budding and release of RCBs (Case II in Figure 1A). Precise imaging analysis using multiple fluorescent markers of chloroplasts or autophagy would be required to demonstrate which event occurs first: the protrusion of a portion of the chloroplast or the association of the autophagic membrane with the chloroplast surface.

In both cases (Case I and Case II in Figure 1A), the autophagic membrane must recognize part of the chloroplast as cargo. Since ATG8-interacting proteins act as a connector between the autophagic membrane and organelles during organelle-selective autophagy in *S. cerevisiae* (Noda et al., 2010), we expect that such proteins also function in target recognition during piecemeal autophagy of chloroplasts. To date, three ATIs have been identified in *Arabidopsis*. As mentioned above, two homologous ATG8-interacting proteins, ATI1 and ATI2, form a type of autophagic vesicle distinct from RCBs (Honig et al., 2012; Michaeli et al., 2014). Another type of ATI termed ATI3 interacts with UBIQUITIN-ASSOCIATED PROTEIN 2a (UBA2a) and UBA2b; these conserved proteins function in ER-associated protein degradation (Zhou et al., 2018). CHARGED MULTIVESICULAR BODY PROTEIN1a (CHMP1a) and CHMP1b are plant ESCRT proteins that are required for transport of RCBs into the vacuole (Spitzer et al., 2015). In the *chmp1a chmp1b* mutant, RCBs accumulate in the cytoplasm, suggesting that these ESCRT proteins mediate the vacuolar sorting of autophagosomes enclosing RCBs rather than the autophagosomal recognition of chloroplasts. How autophagic vesicles interact with parts of chloroplasts during sugar starvation-induced, piecemeal-type chlorophagy remains largely unknown.

## Future Perspectives

Growing evidence suggests that autophagy plays a vital role in helping plants overcome sugar starvation. When photosynthesis is limited due to environmental cues, photosynthetic proteins cannot function and temporarily become extraneous. Thus, perhaps part of the chloroplast stroma is digested as a strategy for adaptation to such suboptimal conditions. Further studies are needed to clarify the mechanism that specifically regulates chloroplast-targeting autophagy, including how morphological changes in chloroplasts are involved in the initiation of autophagy and how autophagic vesicles interact with the target region on the chloroplast surface. The coordination among autophagy and other proteolysis pathways should be further evaluated to increase our understanding of the overall strategy used by plants to adapt to sugar starvation. In this mini-review, we explored the possible roles of autophagy, the ubiquitin proteasome system, and the CV-mediated pathway during dark-induced sugar starvation; however, other components, such as intraplastidic proteases (Nishimura et al., 2017), can contribute to the release of free AAs. Advances in our understanding of these processes would facilitate the development of new ways to improve plant tolerance to abiotic stress by inducing sugar starvation through the modification of multiple proteolytic processes.

## Author Contributions

MI and SN wrote the manuscript. SN designed the figures with the support of MI. NL revised and critically evaluated the manuscript. All authors read and approved the manuscript.

### Conflict of Interest Statement

The authors declare that the research was conducted in the absence of any commercial or financial relationships that could be construed as a potential conflict of interest.
